# A Structured Narrative Review of Contemporary Dietary Strategies Following Acute Coronary Syndromes

**DOI:** 10.3390/jcdd13090414

**Published:** 2026-08-26

**Authors:** Yu Ling Cheung, Christian Hamilton-Craig, Jamie Layland

**Affiliations:** 1 Austin Health, Melbourne, VIC 3084, Australia; 2 Faculty of Medicine, University of Queensland, Brisbane, QLD 4072, Australia; c.hamiltoncraig@uq.edu.au; 3 Department of Cardiology, Peninsula University Hospital, Melbourne, VIC 3199, Australia; jlayland@phcn.vic.gov.au; 4 Peninsula Clinical School, Monash University, Melbourne, VIC 3199, Australia

**Keywords:** acute coronary syndrome, myocardial infarction, nutrition therapy, Mediterranean diet, cardiovascular disease prevention, lifestyle intervention, secondary prevention

## Abstract

Background/Aim: Acute coronary syndromes (ACS) remain a leading cause of morbidity and mortality worldwide. Although a Mediterranean-style diet is recommended for secondary prevention following ACS, patients are increasingly interested in alternative dietary approaches. This structured narrative review critically compares the evidence supporting eight dietary patterns for secondary prevention following ACS. Methods: A structured literature search of PubMed/MEDLINE was performed from database inception through October 2025. Randomized controlled trials evaluating Mediterranean, Dietary Approaches to Stop Hypertension (DASH), plant-based, intermittent fasting, low-carbohydrate/ketogenic, Palaeolithic, Atkins, and Carnivore diets in adults following ACS were prioritised. Where randomized evidence in post-ACS populations was unavailable, evidence from broader cardiovascular populations was included to provide clinical context. Primary outcomes were major adverse cardiovascular events and cardiovascular mortality, while secondary outcomes included low-density lipoprotein cholesterol (LDL-c), blood pressure, body weight, and adherence to dietary interventions. Results: Twenty-eight studies met the inclusion criteria, comprising five Mediterranean-style diet studies, seven DASH studies, four vegetarian/vegan diet studies, four intermittent fasting studies, four low-carbohydrate studies, two Atkins studies, one Palaeolithic diet study, and one Carnivore diet study. Despite the emergence and popularity of other diets, the Mediterranean diet demonstrated the strongest and most consistent evidence for reducing recurrent cardiovascular events and improving cardiovascular risk factors following ACS. DASH and plant-based dietary patterns improved cardiovascular risk factors but lacked robust post-ACS outcome data. Evidence supporting intermittent fasting, low-carbohydrate/ketogenic, Palaeolithic, Atkins, and Carnivore diets was limited, with few randomized studies and insufficient evidence to support their routine use in secondary prevention. Conclusions: A Mediterranean-style diet remains the dietary pattern supported by the strongest evidence for secondary prevention following ACS. Until further pragmatic randomized trials are available, clinicians should continue recommending Mediterranean-style dietary patterns while individualising dietary advice according to patient preferences, cultural practices, and long-term adherence.

## 1. Introduction

Despite advances in medical therapy and revascularisation, acute coronary syndrome (ACS) remains a leading cause of mortality and morbidity worldwide, with survivors at substantial risk of recurrent cardiovascular events [1]. Consequently, secondary prevention strategies are essential to reduce recurrent myocardial infarction (MI), heart failure, and cardiovascular mortality [1,2]. Dietary modification is a cornerstone of secondary prevention and may prevent recurrent cardiovascular events by lowering low-density lipoprotein cholesterol (LDL-c), blood pressure, body weight, and systemic inflammation and improving glycaemic control [1].

Current international cardiovascular guidelines, including those from the European Society of Cardiology and the American Heart Association (AHA)**,** recommend a Mediterranean-style diet as one of the principal dietary approaches for cardiovascular risk reduction following ACS [1,2]. This approach is characterised by a high intake of vegetables, fruits, legumes, whole grains, nuts, extra-virgin olive oil, and fish, while limiting ultra-processed foods, red and processed meats, and saturated fat. Although alcohol intake has historically been included in the Mediterranean diet, current guidelines do not recommend initiating alcohol consumption [1,2].

Large systematic reviews have consistently highlighted the benefits of the Mediterranean diet on lowering cardiovascular events and mortality in primary and secondary prevention settings [3,4]. However, in recent years, patients have increasingly sought advice regarding alternative diets—including low-carbohydrate, Atkins, intermittent fasting, Palaeolithic, and Carnivore diets—which are widely promoted in popular media despite having limited evidence in secondary cardiovascular prevention [5]. To our knowledge, no previous review has comprehensively compared guideline-endorsed Mediterranean-based eating with emerging dietary approaches specifically within the context of secondary prevention following ACS. Given the growing popularity of these dietary patterns and the heterogeneous quality of supporting evidence, an updated synthesis is warranted to inform both clinical practice and future research.

The objective of this structured narrative review was to critically compare the evidence supporting eight contemporary dietary patterns in adults following ACS, with particular emphasis on major adverse cardiovascular events, cardiovascular risk factors, dietary adherence and clinical applicability. By synthesising both guideline-supported and emerging dietary strategies, this review aims to provide clinicians with a practical evidence-based framework for dietary counselling following ACS.

### 1.1. Review Design

This structured narrative review was conducted to evaluate the evidence supporting diets for secondary prevention following ACS. Unlike a systematic review, the purpose of this review was to provide a clinically focused synthesis of the current evidence rather than a comprehensive appraisal of all available studies. Particular emphasis was placed on studies evaluating major adverse cardiovascular events, cardiovascular risk factors, dietary adherence, and the practical feasibility of dietary interventions in patients following ACS. The review was designed to compare dietary patterns that are either recommended by contemporary cardiovascular guidelines or have gained increasing public interest despite limited evidence. These dietary patterns are summarised in Table 1. A structured narrative review was considered the most appropriate methodology because the available evidence comprised heterogeneous dietary interventions, diverse study designs, and relatively few randomized controlled trials for several emerging dietary patterns, which precluded a coherent quantitative synthesis.

### 1.2. Literature Search

**Figure 1:**
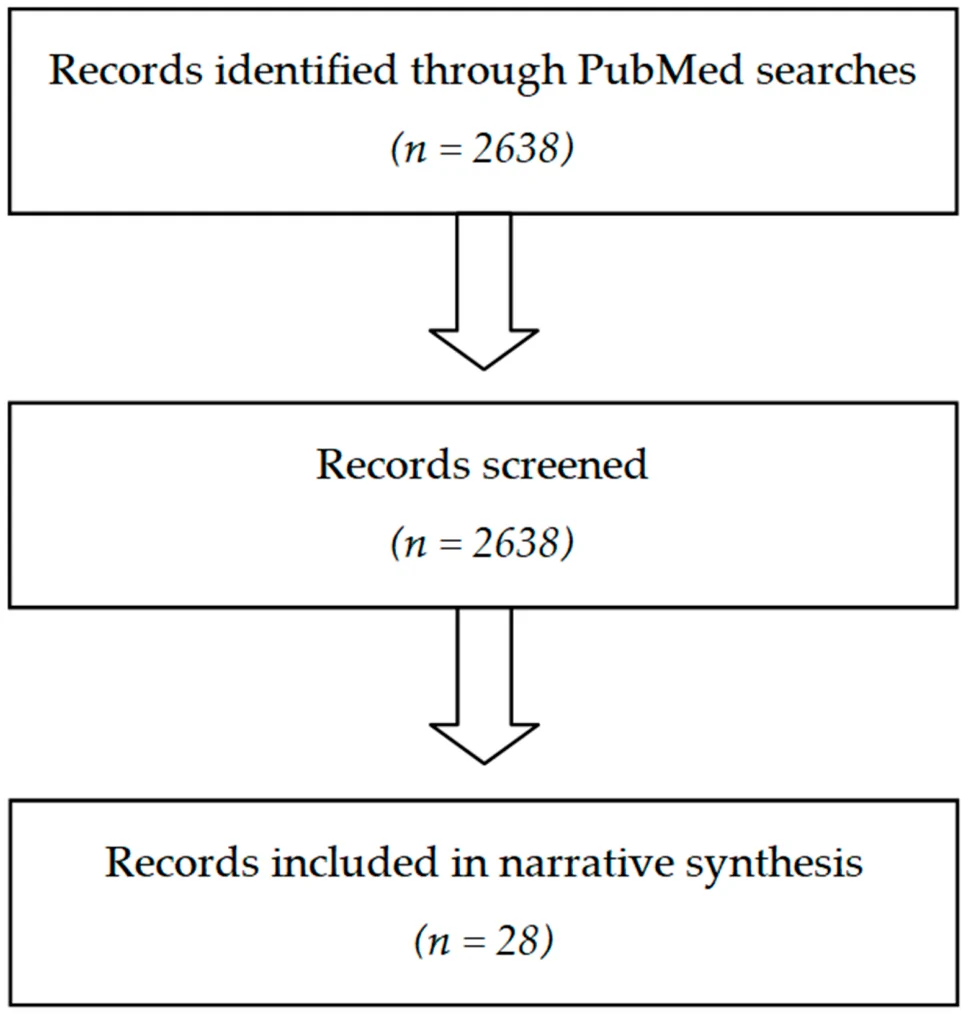
Flow chart of identified, screened, and final studies included in the review.

A literature search was performed using PubMed/MEDLINE from database inception until October 2025. The study identification and selection process is summarised in Figure 1. As this was a structured narrative review rather than a systematic review, a PRISMA flow diagram was not performed. To improve transparency and reproducibility, detailed search strategies, filters applied, and records retrieved are provided in Supplementary Table S1. Reference lists of eligible studies and relevant international cardiovascular guidelines were also manually screened to identify additional relevant publications. The search strategy incorporated combinations of Medical Subject Headings (MeSH) and free-text keywords including “myocardial infarction”, “acute coronary syndrome”, “diet”, “Mediterranean diet”, “Dietary Approaches to Stop Hypertension”, “plant-based diet”, “vegetarian”, “vegan”, “ketogenic”, “low-carbohydrate”, “intermittent fasting”, “Palaeolithic”, “Atkins”, “Carnivore”, “secondary prevention”, and “cardiovascular disease”.

Studies were eligible for inclusion if they:

- Enrolled adults (>18 years) following ACS;
- Evaluated one or more dietary interventions or dietary patterns;
- Reported cardiovascular outcomes, cardiovascular risk factors, or dietary adherence; and
- Were randomized controlled trials where available.

Due to limited evidence specifically evaluating dietary interventions after ACS, studies conducted in broader cardiovascular populations and non-randomized studies were included when directly relevant to secondary prevention and cardiovascular risk modification. For emerging dietary patterns with limited literature (e.g., Carnivore diet), broader search terms were used to maximize identification of potentially relevant studies. Studies were excluded if they were animal studies, case reports, conference abstracts, editorials, non-English publications without an available full text, or unrelated to dietary management following ACS.

Given the heterogeneity of dietary interventions, study designs, outcome measures, and the limited number of randomized trials for several dietary patterns, a formal systematic review and meta-analysis were not performed. Instead, findings were synthesised with particular attention to their methodological quality, potential confounding, and clinical applicability.

### 1.3. Study Selection

Titles and abstracts were screened by one reviewer (YLC) to identify potentially eligible studies. Full-text articles were subsequently assessed for eligibility according to the predefined inclusion and exclusion criteria. Where uncertainty existed regarding study eligibility, relevant clinical guidelines and reference lists were reviewed before a final decision was made.

### 1.4. Selection of Dietary Patterns

The dietary patterns included in this review were selected based on one or more of the following criteria:

- Recommendation by international cardiovascular guidelines;
- Availability of published evidence evaluating cardiovascular outcomes;
- Increasing popularity among patients in clinical practice; and
- Clinical relevance to secondary cardiovascular prevention.

Although dietary patterns such as the Portfolio, Nordic, Mediterranean-DASH Intervention for Neurodegenerative Delay, and Flexitarian diets have demonstrated potential cardiovascular benefits, they were not included because of either substantial overlap with the dietary principles of the Mediterranean, DASH, or plant-based dietary patterns, or the limited availability of evidence specifically evaluating their role following ACS. The selected dietary patterns therefore represent the most clinically relevant and commonly discussed approaches currently encountered in cardiovascular practice.

### 1.5. Evidence Synthesis

Evidence was synthesised according to each dietary pattern. For each dietary approach, study design, participant characteristics, duration of follow-up, cardiovascular outcomes, effects on cardiovascular risk factors, adherence, limitations, and potential sources of bias were critically evaluated. Particular emphasis was placed on the strength and consistency of evidence, clinical applicability following ACS, and areas where evidence remain limited. Given the heterogeneity of the available evidence, randomized controlled trials and studies reporting clinical cardiovascular outcomes were prioritised, while findings from observational studies were interpreted cautiously in light of their inherent risk of residual confounding and selection bias.

### 1.6. Dietary Patterns

1. Mediterranean
2. DASH
3. Vegetarian/vegan
4. Intermittent fasting
5. Low carbohydrate
6. Atkins
7. Palaeolithic
8. Carnivore

## 2. Results

### 2.1. Mediterranean

A Mediterranean-style diet is characterised by a high intake of vegetables, fruits, whole grains, legumes, nuts, fish, and extra-virgin olive oil, while limiting red and processed meats, saturated fat, and ultra-processed foods. It is the dietary pattern most consistently recommended by international cardiovascular guidelines for secondary prevention.

**Figure 2:**
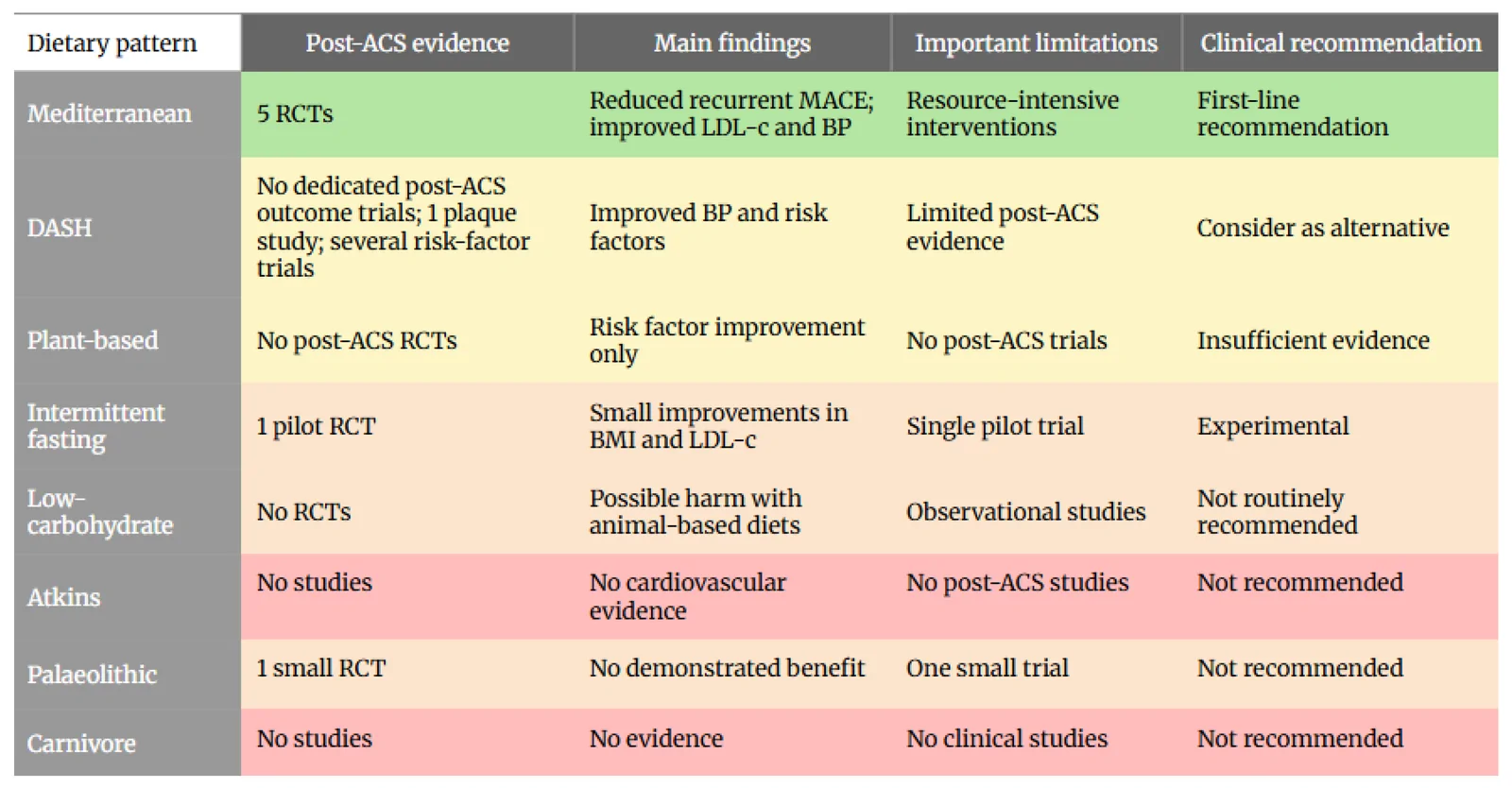
Summary of post-ACS research. The colours correspond to the quality of available evidence (i.e., green indicates the dietary pattern with the strongest supporting post-ACS evidence; yellow indicates the dietary pattern with observed benefits on cardiovascular risk factors only; orange indicates limited evidence on cardiovascular outcomes and risk factors; and red means very limited or no evidence). The intermittent fasting pilot trial was on time-restricted eating. RCTs = randomized controlled trials; MACE = major adverse cardiovascular events; LDL-c = low-density lipoprotein cholesterol; BP = blood pressure; DASH = Dietary Approaches to Stop Hypertension; ACS = acute coronary syndrome; BMI = body mass index.

Five large randomized trials evaluating the Mediterranean or Mediterranean-style diet after ACS were identified. The principal findings are summarised in Figure 2. Overall, these randomized trials consistently demonstrated reductions in recurrent cardiovascular events together with favourable improvements in LDL-c, blood pressure, and body weight. However, most interventions incorporated intensive dietary counselling, behavioural support, or additional lifestyle modifications, making it difficult to isolate the effects of dietary change alone.

The landmark Lyon Diet Heart Study randomized 605 post-MI patients to a Mediterranean-style diet or usual care [7]. After three years, the intervention group experienced a marked reduction in the composite endpoint of cardiac deaths and non-fatal myocardial infarction (hazard ratio 0.28; *p* = 0.0001). Although the reported treatment effect was substantial, relatively few outcome events occurred (6 vs. 19 cardiac deaths; 8 vs. 25 non-fatal MI), increasing uncertainty around the precision of the estimated effect. Furthermore, conventional cardiovascular risk factors changed little between groups, making the biological mechanism underlying the observed reduction in cardiovascular events uncertain [8].

Two South Asian trials also evaluated Mediterranean-style, fibre-rich dietary interventions. The Indian Diet Heart Study randomized patients within 48 h of acute MI or unstable angina to either a plant-rich dietary intervention or a low-fat AHA-recommended diet [9]. The intervention combined dietary modification with smoking cessation, counselling, exercise, and short-term calorie restriction. The intervention group reported fewer cardiac events (50 vs. 82; *p* < 0.001), lower mortality (21 vs. 38; *p* < 0.001), greater weight loss, lower LDL-c, and improved blood pressure after one year. However, because multiple lifestyle interventions were introduced simultaneously, these benefits cannot be attributed to diet alone. In addition, concerns regarding the methodological reliability of this study have been raised [10]. Similarly, the Indo-Mediterranean Diet Heart Study reported fewer MIs and cardiac deaths among participants receiving a Mediterranean-style diet compared with a healthy control diet [11]. However, participants without previous MI were also enrolled, and outcomes were not analysed separately, reducing the applicability of these findings to strictly post-ACS groups.

The Oslo Diet-Heart Study evaluated dietary modification combined with smoking cessation following first MI [12]. The intervention included a diet with Mediterranean-style features, such as increasing fibre and unsaturated fat intake. At the 11-year follow-up, the intervention group experienced fewer fatal MIs and demonstrated a more favourable cardiovascular risk profile, but no significant difference in cardiovascular mortality compared to the control group. Interpretation of these findings is limited by the non-blinded outcome assessment, uncertainty regarding whether dietary changes were maintained beyond the initial years of follow-up, and the substantial contribution of smoking cessation to the observed benefits.

In contrast, the Heart Institute of Spokane Diet Intervention and Evaluation Trial more closely reflected routine clinical practice, as participants prepared their own meals without food provision or intensive dietary support [13]. Participants practicing either a Mediterranean-style diet or a healthy low-fat diet demonstrated similar rates of recurrent cardiovascular events over four years, with no significant differences in cardiovascular risk factors. These findings suggest that the benefits of Mediterranean-style dietary interventions may be attenuated in real-world settings without intensive dietary support.

Taken together, the available evidence consistently favours a Mediterranean-style diet for secondary prevention following ACS. Nevertheless, several important limitations should be acknowledged. Most interventions were multifactorial, incorporating dietary counselling, behavioural support, smoking cessation, exercise, or food provision. Consequently, the impact of dietary change alone cannot be fully established. Furthermore, several landmark studies compared Mediterranean dietary interventions with relatively limited usual care, rather than contemporary guideline-directed secondary prevention, and many were conducted in Mediterranean or highly motivated populations where dietary adherence may not reflect routine clinical practice. Despite these limitations, the consistency of benefit across randomized trials supports a Mediterranean-style diet as the dietary pattern with the strongest evidence base for secondary prevention following ACS.

### 2.2. DASH

The Dietary Approaches to Stop Hypertension (DASH) diet emphasises fruits, vegetables, whole grains, legumes, and low-fat dairy products while limiting sodium, saturated fat, and processed foods [6]. Although the DASH diet is well established for lowering blood pressure [14,15,16,17], evidence supporting its role in secondary prevention following ACS remains limited. Seven relevant studies were identified (Table 1), including one observational study evaluating cardiovascular events, one randomized trial assessing coronary atherosclerosis progression, and five studies examining cardiovascular risk factors. Overall, DASH consistently improved cardiovascular risk factors, particularly blood pressure, but evidence demonstrating reductions in recurrent cardiovascular events following ACS is lacking.

Observational evidence suggests that greater adherence to a DASH-style dietary pattern is associated with lower risks of coronary heart disease (RR 0.76; 95% CI 0.67–0.85) and stroke (RR 0.81; 95% CI 0.71–0.94) [18]. However, this study did not specifically evaluate post-ACS patients and, like all observational studies, remains susceptible to residual confounding and healthy-user bias.

Randomized studies have demonstrated favourable effects on surrogate cardiovascular outcomes. A DASH-based lifestyle intervention significantly reduced non-calcified coronary plaque volume compared with usual care after one year (−51.3 ± 79.5 mm^3^ versus no significant change) [19]. Although these findings suggest that DASH may slow atherosclerosis progression, the study was non-blinded and assessed coronary computed tomography angiography rather than clinical outcomes.

Most randomized DASH trials have consistently demonstrated improvements in blood pressure and other cardiovascular risk factors [14,20,21,22]. These interventions were resource-intensive, incorporating dietary counselling, food provision, exercise, weight loss, smoking cessation, alcohol reduction, and household engagement. In contrast, a pragmatic trial conducted in Hong Kong primary care found that a single dietary counselling session did not significantly improve cardiovascular risk factors after one year [22], suggesting that sustained behavioural support may be necessary to achieve meaningful benefits.

Taken together, the available evidence supports the DASH diet as an effective strategy for improving blood pressure and cardiovascular risk factors. Nevertheless, evidence specifically evaluating recurrent cardiovascular events following ACS remains sparse. Consequently, while DASH represents a reasonable dietary approach for cardiovascular risk reduction, its role in secondary prevention after ACS remains less well established than that of a Mediterranean-style diet.

### 2.3. Vegetarian/Vegan

Plant-based diets encompass a spectrum of eating patterns. Vegetarian diets exclude meat and fish, whereas vegan diets avoid all animal-derived products, including eggs and dairy, and therefore usually require vitamin B12 supplementation [6].

No randomized trials specifically evaluating vegetarian or vegan diets following ACS were identified. Two trials evaluated broader populations with established coronary artery disease (Table 1). Overall, the available evidence is insufficient to determine whether these diets provide additional cardiovascular benefits beyond contemporary Mediterranean-style dietary recommendations for secondary prevention following ACS.

The Ornish Lifestyle Heart Trial evaluated an intensive lifestyle programme involving a low-fat vegetarian diet, smoking cessation, exercise, stress management, and group support in patients with established coronary artery disease [23,24,25]. Although participants demonstrated improvements in coronary atherosclerosis and cardiovascular risk factors, these benefits do not reflect dietary change alone given the multifactorial intervention. The volunteer-based study design further introduces the potential for healthy-user bias, limiting generalisability to routine clinical practice.

The evidence for vegan diets is also limited. A randomized trial involving 100 adults with established coronary artery disease compared a vegan diet with an AHA-recommended diet over eight weeks [26]. The vegan diet was exclusively plant-based, while the AHA diet was largely plant-based with lean protein sources (e.g., fish, lean poultry, low-fat dairy). Both groups demonstrated no changes in cardiovascular risk factors (lipids, weight, blood pressure), with no clear advantage of the vegan intervention, though it is likely that the short intervention period was unlikely to capture meaningful differences in recurrent cardiovascular events or long-term dietary adherence.

Despite the absence of post-ACS trials, vegetarian and vegan dietary patterns have consistently been associated with improvements in body weight, LDL-c, blood pressure, and glycaemic control in broader cardiovascular populations [27]. However, these findings should be interpreted carefully, as most studies evaluated primary prevention or stable coronary artery disease rather than patients recovering from ACS.

Taken together, the current evidence does not support recommending vegetarian or vegan diets over guideline-endorsed Mediterranean dietary patterns for secondary prevention following ACS. Nevertheless, increasing consumption of plant-based foods and dietary fibre aligns with recommendations from most contemporary cardiovascular guidelines [1,2,6]. Pragmatic randomized trials are required to determine whether vegetarian or vegan dietary patterns provide additional benefits following ACS.

### 2.4. Intermittent Fasting

Intermittent fasting alternates periods of fasting with unrestricted food intake. Common regimens include daily 16 h fasts, alternate-day fasts, and fasting on two days per week [6]. Intermittent fasting has gained popularity because it is inexpensive, requires minimal dietary counselling, and does not restrict specific food groups. Although fasting has demonstrated benefits for weight loss and metabolic health [6], it is not well-explored for secondary prevention after ACS. Four relevant studies were identified (Table 1), including one pilot randomized trial in post-MI patients and three studies evaluating weight loss and cardiovascular risk factors in broader populations. Overall, intermittent fasting demonstrated modest improvements in cardiovascular risk factors but had insufficient evidence on its effect on recurrent cardiovascular events following ACS.

The Intermittent Fasting After ST-Segment-Myocardial Infarction trial provides the only direct evidence of intermittent fasting following ACS. Forty-eight patients were randomized within 48 h of ST-elevation MI to either an 8 h daily eating window or unrestricted meal timing [28]. No dietary advice or exercise programme was prescribed, allowing the intervention to more closely reflect the independent effects of meal timing. After six months, each group observed one recurrent non-ST-elevation MI and one cardiac death. Participants assigned to intermittent fasting demonstrated modest improvements in left ventricular ejection fraction (10% vs. 2.5%; *p* = 0.038), body mass index (−1.34 vs. −0.68 kg/m^2^; *p* = 0.047), and LDL-c (−2.0 vs. −1.1 mmol/L; *p* < 0.001), while systolic blood pressure remained unchanged. Adherence was high, with participants fasting on approximately 80% of study days. Although these findings are encouraging, this warrants corroboration in larger trials.

Research in non-ACS populations has primarily focused on weight loss rather than cardiovascular events [29,30,31]. Across these studies, improvements in cardiovascular risk factors and adherence were inconsistent. One randomized trial reported greater attrition with alternate-day fasting compared with continuous calorie restriction after one year (38% vs. 29%) despite full food provision [29], highlighting potential challenges with long-term adherence. In addition, several interventions lasted only three months [30,31], limiting the assessment of long-term cardiovascular benefits. Collectively, these studies suggest that intermittent fasting has uncertain long-term cardiovascular impacts, owing to the short interventions and suboptimal adherence in research settings.

Taken together, intermittent fasting demonstrates promising effects on body weight and selected cardiovascular risk factors, with good short-term adherence observed in the only post-ACS trial. However, the evidence on long-term cardiovascular events following ACS remains unclear. Larger pragmatic randomized trials with longer follow-up are required before intermittent fasting can be considered for secondary prevention after ACS.

### 2.5. Low-Carbohydrate Diets

Low-carbohydrate diets restrict carbohydrate intake with the aim of promoting weight loss and improving glycaemic control [6]. The ketogenic diet is an extreme form of carbohydrate restriction in which dietary fat—commonly derived from foods such as meat, eggs, full-fat dairy, oils, nuts, and fatty fish—comprises more than 60% of total energy intake. Carbohydrate restriction can promote ketone production from fatty acids, providing an alternative energy source during periods of reduced glucose availability.

Evidence evaluating low-carbohydrate diets following ACS is limited and consists predominantly of observational studies. Four relevant studies were identified (Table 1), including one observational study in MI survivors, two observational studies in broader cardiovascular populations, and one prospective study evaluating coronary atherosclerosis. Overall, the current evidence suggests that low-carbohydrate approaches have minimal apparent benefits on cardiovascular health, with potential harms from animal-based versions.

Among 4098 MI survivors from the Nurses’ Health Study and Health Professionals Follow-up Study, greater adherence to an animal-based low-carbohydrate diet was associated with higher all-cause (HR 1.33) and cardiovascular mortality (HR 1.51) when comparing the highest and lowest quintiles [32]. This association disappeared after adjusting for saturated fat intake, suggesting that dietary composition rather than carbohydrate restriction itself may explain the observed risk. Conversely, plant-based low-carbohydrate diets were not associated with increased mortality.

Similar findings were observed in broader cardiovascular populations. A separate analysis of 82,802 women without established cardiovascular disease found no association between overall low-carbohydrate dietary patterns and incident coronary heart disease, although greater consumption of plant-derived fats and proteins was associated with lower cardiovascular risk (HR 0.70; 95% CI, 0.56–0.88; *p* = 0.002) [33]. Likewise, a prospective study of 5115 young adults demonstrated slower progression of coronary artery calcium among individuals consuming higher-carbohydrate diets, whereas animal-based low-carbohydrate diets were associated with more rapid progression [34].

While appearing to favour plant-based low-carbohydrate diets, these findings should be interpreted considering various limitations. All major outcome studies were observational and are susceptible to residual confounding, healthy-user bias, and dietary misclassification associated with food frequency questionnaires. Consequently, the observed associations should not be attributed solely to carbohydrate restriction but also to potential differences in dietary quality and lifestyle behaviours.

The recent KETO-CTA prospective trial investigated coronary plaque progression among metabolically healthy adults following a ketogenic diet but was later retracted due to methodological concerns [35,36]. A sub-study matching KETO-CTA participants with matched controls did not demonstrate significant differences in coronary artery calcium or total plaque burden after one year on a high-fat ketogenic diet [37]. However, these results should be interpreted cautiously, as participants were young, healthy, and do not represent the high-risk patients recovering from ACS.

Taken together, there is insufficient evidence to recommend low-carbohydrate or ketogenic dietary patterns for secondary prevention following ACS. While carbohydrate restriction may improve glycaemic control and facilitate weight loss [38], available evidence suggests that animal-based low-carbohydrate dietary patterns might be associated with poorer cardiovascular outcomes than plant-based approaches. Practically speaking, these diets may garner lower adherence given their restrictive nature, financial cost, and exclusion of carbohydrate-rich foods that form the basis of many traditional dietary patterns.

### 2.6. Atkins

The Atkins diet is a commercially developed low-carbohydrate dietary pattern that promotes unrestricted intake of protein and fat during its initial phases while substantially restricting carbohydrate consumption [39]. Although widely promoted for weight loss, its role in secondary prevention following ACS has not been investigated.

No post-ACS studies evaluating the Atkins diet were identified (Table 1). Evidence from weight-loss trials suggests that long-term adherence is poor, with attrition approaching 50% after two years, limiting its clinical applicability [39,40]. Acknowledging the lack of cardiovascular outcome data, there is currently insufficient evidence to recommend the Atkins diet for secondary prevention following ACS.

### 2.7. Palaeolithic

The Palaeolithic diet aims to replicate the presumed dietary patterns of ancestral hunter-gatherers through promoting fruits, vegetables, nuts, lean meats, fish, and other minimally processed foods while excluding grains, legumes, dairy products, refined sugars, and ultra-processed foods [6]. Unlike ketogenic or low-carbohydrate diets, the Palaeolithic diet focuses on food quality rather than specific macronutrient targets.

There is very limited post-ACS research on the Palaeolithic diet. Only one randomized trial was identified (Table 1), with no evident improvements in recurrent cardiovascular events. 19 men with ischaemic heart disease were instructed to follow either a Palaeolithic or Mediterranean diet for 12 weeks [41]. Neither group saw significant differences in body weight or blood pressure at follow-up, although the short intervention and small sample size likely limited the trial’s ability to detect clinically meaningful differences in cardiovascular risk factors or recurrent cardiovascular events.

As such, the effectiveness of the Palaeolithic diet for secondary prevention following ACS remains uncertain. In addition to the limited evidence base, the diet may present practical challenges because of its restrictions, financial costs, and exclusion of commonly consumed food groups such as grains, legumes, and dairy products, which may reduce long-term adherence, particularly in lower resource settings [42].

Taken together, there is insufficient evidence to recommend the Palaeolithic diet for secondary prevention following ACS. Larger pragmatic randomized trials with longer follow-up are required before its role can be established.

### 2.8. Carnivore

The Carnivore diet is an extreme diet consisting almost exclusively of animal-derived foods while excluding fruits, vegetables, grains, legumes, and other plant-based foods [43]. Despite increasing popularity in online communities, no clinical trials have evaluated the Carnivore diet in post-ACS patients (Table 1). The current evidence is limited to an indirect observational report that described a marked elevation in LDL-c among self-reported Carnivore users [43]. A major disadvantage of the Carnivore diet is that it directly contradicts contemporary cardiovascular guidelines in its exclusion of fibre-rich plant foods [1,2] and excessive intakes of red and processed meat, at the detriment of cardiovascular health [44].

Taken together, there is currently no evidence supporting the Carnivore diet for secondary prevention following ACS. Given its highly restrictive nature, potential adverse effects on LDL-c, and lack of supporting clinical evidence, clinicians should discourage its routine use in patients with established cardiovascular disease until robust prospective studies become available.

## 3. Discussion

### 3.1. Principal Findings

**Figure 3:**
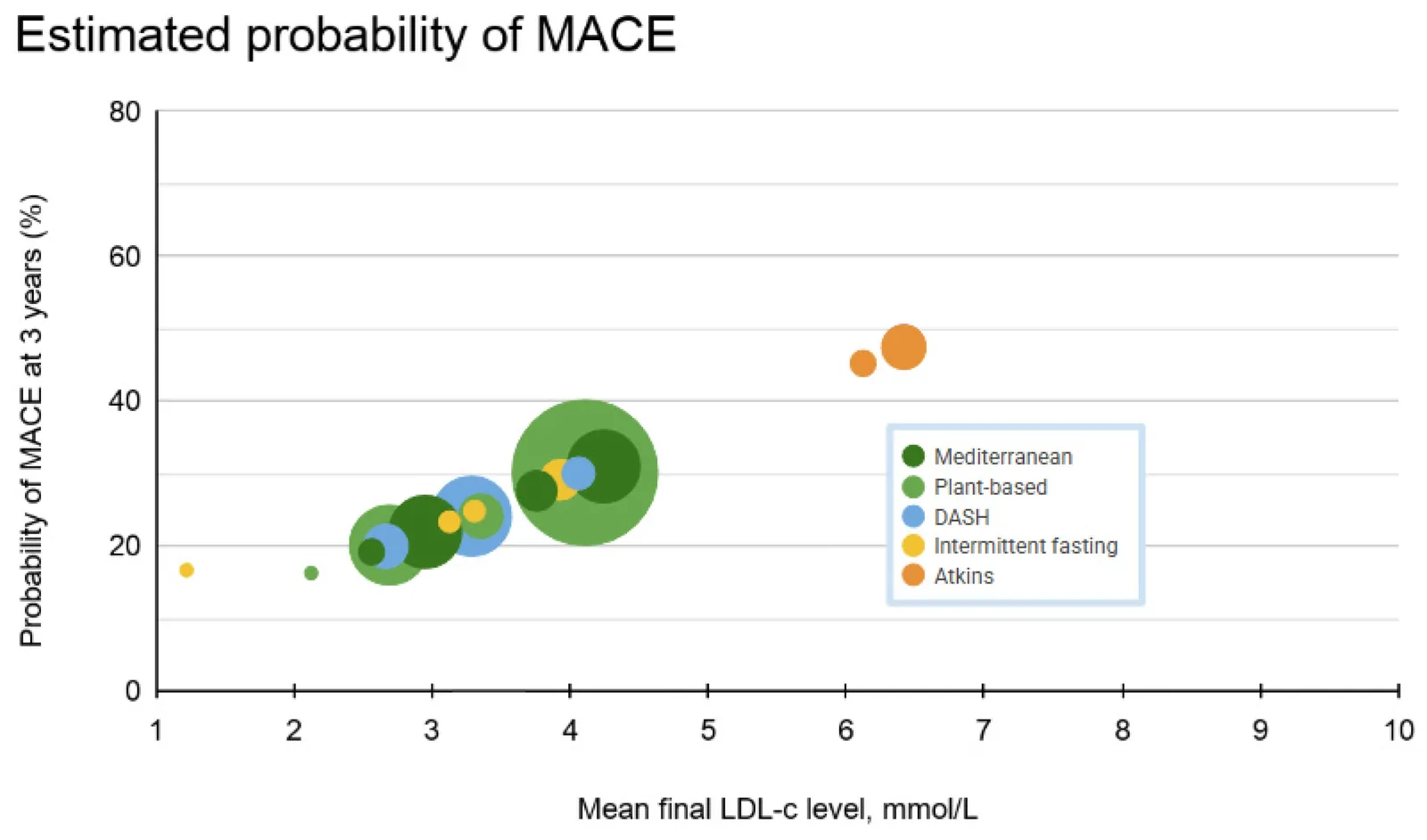
Schematic diagram of the mean final LDL-c values reported by included randomized trials. Non-randomized trials were excluded. The size of the bubble reflects the number of participants following each dietary intervention. Low-carbohydrate and Carnivore diets did not have randomized data, and the Palaeolithic trial did not measure LDL-c; thus these three diets were not displayed.

The present narrative review found that a Mediterranean-style diet remains the dietary pattern supported by the strongest evidence for secondary prevention following ACS. Randomized trials and long-term observational studies have demonstrated positive effects on adverse cardiovascular events, lipid profiles, blood pressure, and cardiovascular mortality. In contrast, evidence supporting alternative dietary patterns—including low-carbohydrate, Atkins, Palaeolithic, Carnivore, and intermittent fasting diets—remains limited, with few studies specifically evaluating post-ACS populations. Their long-term efficacy and safety for secondary prevention remain unclear. A summary of the evidence supporting each dietary pattern is provided in Table 1. Figure 3 visualises the final LDL-c reported in the included randomized trials. It is important to acknowledge that many landmark Mediterranean diet trials, including the Lyon Diet Heart Study, delivered dietary interventions with regular behavioural support, regular dietary counselling, and, in some studies, food provision. These interventions may have enhanced dietary adherence and the benefits seen, potentially limiting feasibility in routine clinical practice. Nevertheless, comparable high-quality evidence is largely lacking for other dietary approaches.

### 3.2. Strengths and Limitations

This review has several strengths. It compares multiple dietary patterns using a structured search strategy while focusing specifically on secondary prevention following ACS, an area with limited research.

Several limitations should be acknowledged. As a narrative review, this paper is inherently susceptible to selection bias and does not include a formal risk-of-bias assessment. A systematic review would provide a higher level of evidence, and therefore the conclusions of this narrative review should be interpreted accordingly. The available evidence for many dietary patterns was limited and heterogeneous, making direct comparison challenging. Furthermore, many dietary interventions included behavioural support or lifestyle changes, making it difficult to isolate the effects of diet alone.

### 3.3. Comparison with Previous Reviews

Prior systematic reviews have consistently demonstrated the cardiovascular benefits of Mediterranean-based dietary patterns across broad populations with cardiovascular disease [3,4]. However, these reviews primarily evaluated the Mediterranean diet in isolation and did not compare it directly with newer dietary patterns. Our review extends the existing literature by critically evaluating both guideline-supported dietary patterns and increasingly popular alternative diets within the context of secondary prevention following ACS. This comparison is clinically relevant, as patients increasingly seek advice regarding dietary patterns promoted through social media and commercial sources despite limited supporting evidence.

### 3.4. Biological Mechanisms

Diet influences cardiovascular health through an interplay of biological pathways, including lipid metabolism, inflammation, endothelial function, and gut microbiome. These effects are likely mediated by not only macronutrient composition but also food quality, nutrient density, and individual metabolic responses. Therefore, evaluating dietary patterns rather than isolated nutrients alone is important when considering cardiovascular disease prevention [45].

Dietary patterns rich in fruits, vegetables, legumes, whole grains, nuts, and extra-virgin olive oil provide dietary fibre, polyphenols, antioxidants, and unsaturated fatty acids [46]. These nutrients may improve lipid metabolism, endothelial function, glycaemic control, and blood pressure while reducing oxidative stress and systemic inflammation [46]. Conversely, dietary patterns characterised by high intakes of saturated fat, processed red meat, and ultra-processed foods may promote harmful metabolic pathways, including increased oxidative stress, LDL-c concentrations, and endothelial dysfunction [46,47]. These foods may also promote the production of pro-atherogenic metabolites such as trimethylamine-N-oxide through interactions with the gut microbiome, which may further drive atherosclerosis and recurrent cardiovascular events [46,47].

Beyond traditional biomarkers, emerging metabolomic research may provide additional insight into the complex biological effects of diet and explain the variable metabolic responses to foods between individuals [48]. Future research may utilise metabolomic signatures alongside microbiome profiling to better understand individual responses to dietary interventions and support more personalised nutritional recommendations following ACS [48].

### 3.5. Practical Implications

Based on the current evidence, clinicians should continue to recommend a diet rich in unprocessed plant-based foods and monounsaturated fat and low in red meat, saturated fat, and processed foods for high-risk post-ACS patients. Dietary recommendations should ideally be tailored to each individual, based on their preferences, cultural background, and resources. For example, citizens in countries with high meat consumption (e.g., Eastern Europe, South America) may benefit more from consuming less meat rather than increasing their intake of antioxidants and unsaturated fat [49]. Similarly, advice for people from low-income communities where diet quality is generally poorer may instead focus on encouraging whole foods and home-cooked meals over ultra-processed foods [50]. Calorierestriction in overweight or obese individuals to help achieve a healthy weight can also help decrease cardiovascular risk and subsequently adverse cardiovascular events.

Although dietary recommendations should be personalised, clinicians should be cautious when endorsing alternative diets that have not been adequately evaluated in post-ACS populations. Patients expressing interest in these approaches should be counselled regarding the limited available evidence and the uncertainty surrounding long-term cardiovascular outcomes.

### 3.6. Future Research

Future research should prioritise adequately powered randomized controlled trials comparing contemporary diets in patients following ACS. Pragmatic, cost-effective trial designs that reflect routine clinical practice are particularly important to improve generalisability and long-term adherence. Alongside traditional cardiovascular outcomes, future studies should evaluate dietary adherence, inflammatory biomarkers, endothelial function, coronary plaque progression, gut microbiome, and metabolomic profiles.

## 4. Conclusions

A Mediterranean-based diet remains the dietary pattern supported by the strongest evidence for secondary prevention following ACS. Evidence supporting DASH, plant-based diets, intermittent fasting, and other emerging dietary approaches remains limited, particularly for recurrent cardiovascular events. Until further pragmatic randomized trials become available, clinicians should continue recommending Mediterranean-style dietary patterns while individualising dietary advice according to patient preferences, culture, and long-term adherence.

## Figures and Tables

**Table 1:** Description of selected dietary patterns [6].

Diets	Description
Mediterranean	A plant-rich dietary pattern emphasising vegetables, fruits, legumes, whole grains, nuts, extra-virgin olive oil, and fish, with moderate fat intake derived largely from monounsaturated fats. Limited red and processed meats, saturated fat, and ultra-processed foods. Alcohol is not required.
DASH	Dietary Approaches to Stop Hypertension (DASH). A dietary pattern rich in fruits, vegetables, whole grains, legumes, nuts, and low-fat dairy, while limiting sodium, saturated fat, added sugars, and processed foods.
Vegetarian/vegan	An umbrella term encompassing vegetarian and vegan dietary patterns. Vegetarian diets exclude meat and fish, whereas vegan diets exclude all animal-derived foods, including dairy and eggs. When appropriately planned, these diets are rich in plant foods, fibre, and unsaturated fats. Vitamin B12 supplementation is generally recommended for vegan diets.
Intermittent fasting	An eating pattern alternating periods of fasting with unrestricted eating. Common approaches include time-restricted eating, alternate-day fasting, and fasting on two days per week. There are no caloric or macronutrient restrictions.
Low-carbohydrate (including ketogenic)	Low-carbohydrate diets restrict carbohydrate intake to varying degrees. Ketogenic diets represent the most restrictive form, typically providing <10% of total energy from carbohydrate and >60% from fat, with the aim of achieving nutritional ketosis. Fat sources may include animal- and plant-derived foods.
Atkins	A commercial low-carbohydrate dietary pattern introduced in the 1960s. Carbohydrate intake is initially severely restricted before gradually increased through successive phases. The diet generally encourages liberal intake of animal-based protein and fat.
Palaeolithic	A dietary pattern modelled on presumed ancestral hunter-gatherer eating habits. Emphasises fruits, vegetables, nuts, seeds, lean meats, and fish while excluding grains, legumes, dairy products, refined sugars, and most processed foods.
Carnivore	An animal-based dietary pattern consisting almost exclusively of meat and other animal-derived foods, including eggs and some dairy products depending on the specific variation. Plant foods are generally excluded.

## Data Availability

The raw data supporting the conclusions of this article will be made available by the authors on request.

## Supplementary Materials

The following supporting information can be downloaded at https://www.mdpi.com/article/10.3390/jcdd13090414/s1. Table S1. PubMed search strategies and search results for dietary patterns included in the structured narrative review.

## Abbreviations

Abbreviation	Definition
ACS	Acute coronary syndrome
AHA	American Heart Association
DASH	Dietary approaches to stop hypertension
LDL-c	Low-density lipoprotein cholesterol
MI	Myocardial infarction
