# Supplementary material for: A Structured Narrative Review of Contemporary Dietary Strategies Following Acute Coronary Syndromes
Source: J Cardiovasc Dev Dis. 2026 Aug 26;13(9):414. doi: 10.3390/jcdd13090414 (PMC13607391; doi:10.3390/jcdd13090414)
Supplement: Supplementary file 1 [file jcdd-13-00414-s001.zip › jcdd-4412820-supplementary.pdf]

Dietary pattern	PubMed search strategy	Filters applied	Date searched	Records retrieved
Mediterranean diet	("Mediterranean diet") AND (Cardiovascular OR MI OR "myocardial infarction" OR "acute coronary syndrome" OR "secondary prevention" OR "coronary artery disease" OR "ischaemic heart disease" OR atherosclerosis)	Adults (≥19 years); Clinical Trial; Randomized Controlled Trial; Observational Study	Oct 2025	622
DASH diet	("DASH diet" OR "dietary approaches to stop hypertension") AND (Cardiovascular OR "myocardial infarction" OR MI OR "acute coronary syndrome" OR "secondary prevention" OR "coronary artery disease" OR "ischaemic heart disease" OR atherosclerosis)	Adults (≥19 years); Clinical Trial; Randomized Controlled Trial; Observational Study	Oct 2025	107
Plant-based diet (vegetarian and vegan diets)	("Diet, Vegetarian"[Mesh] OR "Diet, Vegan"[Mesh] OR plant-based OR plant-food) AND (Cardiovascular OR "myocardial infarction" OR MI OR "acute coronary syndrome" OR "secondary prevention" OR "coronary artery disease" OR "ischaemic heart disease" OR atherosclerosis)	Adults (≥19 years); Clinical Trial; Randomized Controlled Trial; Observational Study	Oct 2025	174
Intermittent fasting	("intermittent fasting" OR "intermittent energy restriction" OR "periodic fasting" OR "alternate-day fasting" OR "alternate day fasting" OR "modified alternate-day fasting" OR "modified alternate day fasting" OR "time-restricted feeding" OR "time restricted feeding" OR "whole day fasting" OR Ramadan OR Ramadhan) AND (Cardiovascular OR "myocardial infarction" OR MI OR "acute coronary	Adults (≥19 years); Clinical Trial; Randomized Controlled Trial; Observational Study	Oct 2025	121

	syndrome" OR "secondary prevention" OR "coronary artery disease" OR "ischaemic heart disease" OR atherosclerosis)			
Low-carbohydrate diet / Atkins diet	(Ketogenic OR Atkins OR low-carb* OR "low carb*" OR high-fat OR LCHF) AND (Cardiovascular OR "myocardial infarction" OR MI OR "acute coronary syndrome" OR "secondary prevention" OR "coronary artery disease" OR "ischaemic heart disease" OR atherosclerosis)	Adults (≥19 years); Clinical Trial; Randomized Controlled Trial; Observational Study	Oct 2025	723
Paleolithic diet	("Diet, Paleolithic"[Mesh] OR paleolithic OR palaeolithic OR paleolithic-type OR paleonutrition OR palaeonutrition OR paleo OR "caveman diet" OR "stone age diet" OR "hunter gatherer diet" OR "primal diet" OR "evolution diet" OR "ancestral human diet" OR "primitive diet") AND (Cardiovascular OR "myocardial infarction" OR MI OR "acute coronary syndrome" OR "secondary prevention" OR "coronary artery disease" OR "ischaemic heart disease" OR atherosclerosis)	Adults (≥19 years); Clinical Trial; Randomized Controlled Trial; Observational Study	Oct 2025	410
Carnivore diet	("Carnivore Diet" OR high-protein OR meat OR "animal-based") AND (Cardiovascular OR Heart)	Adults (≥19 years); Clinical Trial; Randomized Controlled Trial; Observational Study	Oct 2025	481

Supplementary Table S1. PubMed search strategies and search results for dietary patterns included in the structured narrative review.
